# The Possible Involvement of Glucagon-like Peptide-2 in the Regulation of Food Intake through the Gut–Brain Axis

**DOI:** 10.3390/nu16183069

**Published:** 2024-09-11

**Authors:** Maria Caterina Baccari, Maria Giuliana Vannucchi, Eglantina Idrizaj

**Affiliations:** 1 Department of Experimental & Clinical Medicine, Section of Physiological Sciences, University of Florence, 50134 Florence, Italy; mcaterina.baccari@unifi.it; 2 Department of Experimental & Clinical Medicine, Research Unit of Histology & Embryology, University of Florence, 50139 Florence, Italy; mariagiuliana.vannucchi@unifi.it

**Keywords:** glucagon-like peptide-2 (GLP-2), gastric motility, food intake, peripheral satiety signals, gut–brain axis

## Abstract

Food intake regulation is a complex mechanism involving the interaction between central and peripheral structures. Among the latter, the gastrointestinal tract represents one of the main sources of both nervous and hormonal signals, which reach the central nervous system that integrates them and sends the resulting information downstream to effector organs involved in energy homeostasis. Gut hormones released by nutrient-sensing enteroendocrine cells can send signals to central structures involved in the regulation of food intake through more than one mechanism. One of these is through the modulation of gastric motor phenomena known to be a source of peripheral satiety signals. In the present review, our attention will be focused on the ability of the glucagon-like peptide 2 (GLP-2) hormone to modulate gastrointestinal motor activity and discuss how its effects could be related to peripheral satiety signals generated in the stomach and involved in the regulation of food intake through the gut–brain axis. A better understanding of the possible role of GLP-2 in regulating food intake through the gut–brain axis could represent a starting point for the development of new strategies to treat some pathological conditions, such as obesity.

## 1. Introduction

The regulation of the hunger-satiety cycle is a complex mechanism of interplay among signals originating from both central and peripheral structures that are mainly integrated at the hypothalamic level [1]. In particular, in the arcuate nucleus, two neuronal populations that exert antagonistic functions in the control of food intake and energy balance have been described: one co-expressing Neuropeptide Y (NPY) and Agouti-related protein (AgRP), and the other co-expressing pro-opiomelanocortin (POMC) and cocaine- and amphetamine-regulated transcript (CART). These neuronal populations express several types of receptors whose activation might cause orexigenic or anorexigenic effects [2,3]. Peptides synthesized by such neuronal populations have been considered essential biomarkers of metabolic disorders occurring in obese subjects, while disruption of these neurons is considered one of the causes of obesity [4]. The arcuate hypothalamic nucleus is recognized as the main integrative center of signals coming from other hypothalamic areas involved in feeding behavior or from extra hypothalamic nuclei [5], such as the nucleus tractus solitarius (NTS), as well as from the periphery. Hypothalamic integration of information from the pancreas, adipose tissue, and gastrointestinal tract plays a key role in the neuroendocrine control of food intake, translating this into a feeding behavior [3,6,7,8,9]. 

The gastrointestinal tract represents also a source of both nervous and hormonal signals which play important roles in the peripheral regulation of the hunger-satiety cycle [9,10,11,12]. Several hormones released by nutrient-sensing enteroendocrine cells can send anorexigenic signals to central structures involved in the regulation of food intake by engaging more than one mechanism [9,13]. Many peptide hormones, entering the systemic circulation, can directly reach the hypothalamic arcuate nucleus, likely through an incomplete blood–brain barrier [14]. Therefore, they may exert their effects through the direct activation of POMC/CART neurons and/or inactivation of NPY/AgRP ones, on which the presence of specific receptors has been identified [3,15]. Furthermore, as better described below, the hormones of intestinal origin can send signals to the central structures involved in the regulation of food intake through the activation of the gut–brain axis. Finally, the observation that some of the intestinal hormones that influence the hunger-satiety cycle by acting at a central level also influence the gastric motor responses responsible for peripheral satiety signals is an interesting feature since peripheral effects may represent additional mechanisms to the central ones in regulating food intake. While this double mechanism has been recognized for some gut-derived anorexigenic hormones (e.g., GLP-1, CCK, and PYY), it has not been completely clarified for GLP-2, which has demonstrated the ability to induce gastrointestinal motor changes in addition to its central anorexigenic effects [16]. 

In the present review, our attention will be focused on the ability of GLP-2 to modulate gastrointestinal motor responses and discuss how its effects could be related to peripheral satiety signals originating from the stomach and involving the central structures regulating food intake through the gut–brain axis. A better understanding of the possible role of GLP-2 in influencing food intake through the gut–brain axis could provide insights into the development of new therapeutic approaches in the treatment of some pathological conditions, such as obesity. In fact, some forms of obesity, characterized by dysregulation of the hunger-satiety cycle, are imputable to alterations of peripheral signaling that promote hyperphagia and weight gain [17] and are susceptible to bariatric therapy as a result [18]. 

## 2. Mechanisms through Which Gut-Derived Hormones May Activate the Gut–Brain Axis to Generate Satiety Signals

In addition to their direct action on receptors in the central nervous system (CNS) via the classic bloodstream way, gut-derived hormones can indirectly send anorexigenic signals to the hypothalamic nuclei, activating vagal afferent fibers from the gastrointestinal tract [18]. The latter mechanism implies the presence of specific receptors on the terminal afferent vagal fibers located in the gut mucosa which have been described for many different regulatory peptides [19]. These signals reach the hypothalamus through the interposition of the NTS [14]. A further mechanism involving the gut–brain axis is represented by the ability of peptide hormones of intestinal origin to exert their anorexigenic effects through the modulation of those gastric motor phenomena known to be a source of peripheral signals involved in the control of food intake at a central level [18,20]. Both gastric accommodation and gastric emptying play an important role in the regulation of organ distension [21]. Gastric wall distension, by causing stretch and tension, stimulates the mechanosensitive receptors which, in turn, activate the vagal afferent nerve fibers. These latter induce satiety signals to the hypothalamic regions involved in the regulation of food intake, through the interposition of the NTS [9,22]. 

Delayed gastric emptying, which plays a critical role in regulating short-term food intake, has been reported to be associated with increasing sensation of satiety and stopping food consumption in humans [23] due to gastric wall distension. This information fits well with the observation that obese subjects present with faster gastric emptying [24,25]. Moreover, gastric emptying determines the rate of arrival of nutrients in the small intestine which, in turn, regulates satiety. As gastric emptying occurs, chyme enters the small intestine where the presence of nutrients is mainly detected by specialized receptors expressed on the apical side of open-type enteroendocrine cells which respond by releasing hormones [26,27,28]. Some anorexigenic hormones reach the stomach to slow gastric emptying, inducing gastric distension and thus contributing to a satiety sensation [29]. Furthermore, some hormones, in response to the arrival of nutrients, mainly lipids and proteins, reach the ileal portion and inhibit its motor responses. This generates a feedback response at the stomach level to induce delayed gastric emptying, a mechanism that is known as the physiological ileal brake reflex [18]. The latter is the result of the activation of enteroendocrine cells and mucosal afferent nerves and is regulated by hormones released from either the proximal gut portion, such as GLP-1 and CCK, or the distal one, as PYY [8]. 

**Figure 1:**
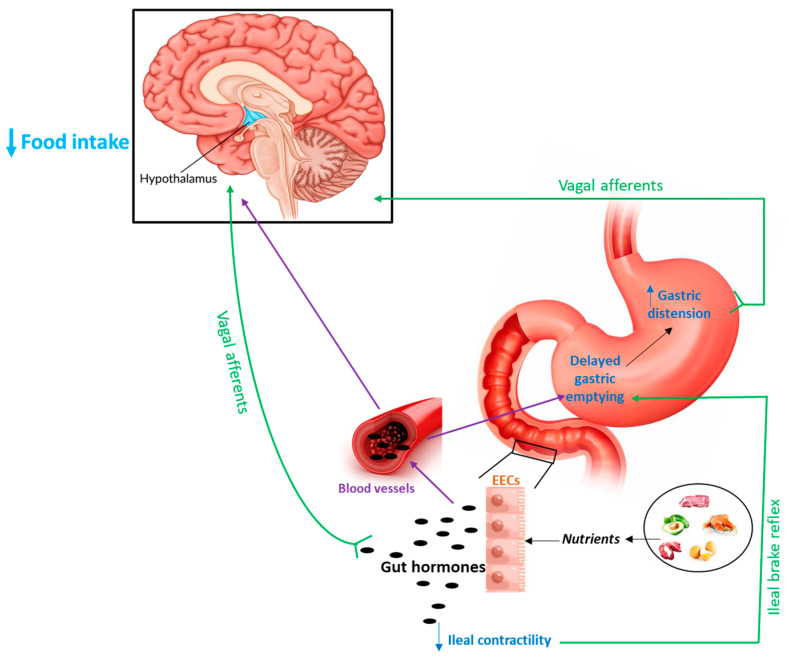
Schematic representation of the main mechanisms through which gut hormones may influence hypothalamic structures to induce anorexigenic effects. Purple lines (bloodstream); green lines (nervous fibers); and EECs (enteroendocrine cells).

An illustration summarizing the main mechanisms through which gut hormones may regulate food intake to induce anorexigenic effects is reported in Figure 1. 

Therefore, the action of intestinal anorexigenic hormones in regulating food intake can be exerted directly at the central level or indirectly at the peripheral level through changes in gastric motor responses which contribute to the sense of satiety. In this view, GLP-2 has been reported to exert some central anorexigenic effects and also to influence gastrointestinal motor responses which could be associated with peripheral satiety signals generated by the stomach and involved in the regulation of food intake, as discussed below.

## 3. Glucagon-Like Peptide 2

**Figure 2:**
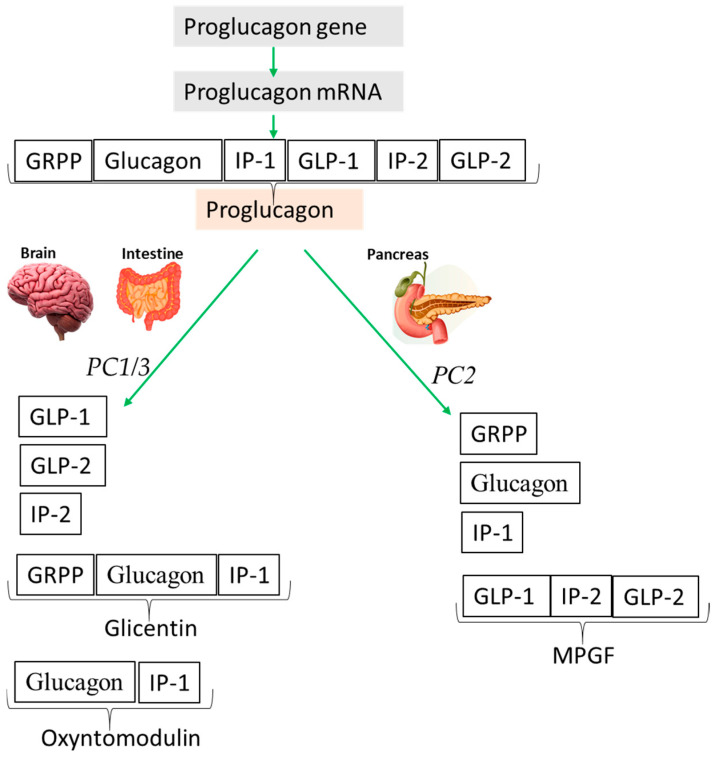
Schematic representation of tissue-specific proglucagon processing in the intestine, brain, and pancreas. The proglucagon gene is transcribed to generate proglucagon messenger RNA (mRNA), which is subsequently translated to the precursor protein, proglucagon.

Glucagon-like peptide 2 and glucagon-like peptide 1 (GLP-1) derive from proglucagon, which is a 158 amino acid precursor protein predominantly expressed in the pancreas, gut, and distinct neuronal populations of the hindbrain [30]. In the brain and the intestine, proglucagon is cleaved by the action of the prohormone convertase 1/3 (PC1/3) into GLP-1, GLP-2, glicentin, glicentin-related polypeptide (GRPP), and oxyntomodulin (OXM) [30,31,32]. A schematic overview of tissue-specific proglucagon processing in the gut, brain, and pancreas is reported in Figure 2.

In the brain and enteroendocrine intestinal L-cells, proglucagon is processed by prohormone convertase 1/3 (PC1/3) to generate glucagon-like peptides-1 (GLP-1) and -2 (GLP-2), intervening peptide-2 (IP-2), glicentin, and oxyntomodulin. 

In pancreatic alpha-cells, proglucagon is processed into glicentin-related pancreatic polypeptide (GRPP), glucagon, intervening peptide 1 (IP1), and major proglucagon fragment (MPGF) by the processing enzyme prohormone convertase 2 (PC2).

GLP-2 is a 33 amino acid peptide mainly expressed in the gut, together with GLP-1, by enteroendocrine L-type cells of the distal small intestine and colonic mucosa [30,33,34,35]. In this regard, the classical definition of L cells as a homogeneous population has recently been revised [36] as important differences between them have been reported along the length of the intestine and also between different species. Furthermore, some subpopulations of L-cells express other peptides in addition to glucagon-like ones [27]. Enteroendocrine L-type cells are activated in response to luminal nutrient content [37], mostly fat and glucose [38,39]. However, there are also several regulating mechanisms underlying L cell secretion, such as circulating hormones (e.g., CCK and some adipokines), paracrine/neuronal substances, as well as gut microbiota with its metabolites [14,27]. The same diet nutrients may cause changes in the gut microbiota composition which in turn influences gut anorexigenic hormone release, thus influencing food intake [40].

GLP-2 has been reported to colocalize with GLP-1 in the same mammalian secretory granule [41] from which it is co-secreted in a 1:1 ratio [42] and the molecular mechanisms that link hormone exocytosis to the circulating patterns of glucagon-like peptides are only recently beginning to be fully understood [27]. Despite the distal localization of L-cells, GLP-1 and GLP-2 plasma levels (as well as other L-cell-derived hormones) rapidly rise following ingestion [43], suggesting the existence of a proximal gut signal also regulating hormone release from the L cells of the distal small intestine [43]. Circulating GLP-2 and GLP-1 are quickly degraded by dipeptidyl peptidase IV (DPP-IV) [44,45], resulting in half-lives of ∼7 and 1–2 min, respectively [30,46,47,48]. Due to their short circulating half-life, the native glucagon-like peptides have only limited pharmacological potential [49], so DPP-IV-resistant analogs as well as DPP-IV inhibitors have been considered for possible therapeutic strategies [50,51]. In this view, GLP-2 analogs have demonstrated their efficacy in the management of short small bowel syndrome (SBS) [52,53,54].

In addition to intestinal L-cells, GLP-2 is also secreted by brainstem neurons that innervate the paraventricular nucleus (PVN) and dorsomedial hypothalamus (DMH) [55]. Moreover, the presence of GLP-2 immunoreactive fibers has also been revealed in the arcuate nucleus (ARC) and PVN [56], hypothalamic areas involved in the regulation of food intake and energy balance, thus suggesting a role for GLP-2 as a neurotransmitter in these areas.

The expression of the specific G protein-coupled GLP-2 receptor (GLP-2R) is predominant in the gastrointestinal tract and CNS of humans and rodents. Its presence has been widely described in the DMH and ARC and particularly in a rodent subpopulation of POMC-expressing neurons [3,29,57] that are known to be implicated in the regulation of energy balance by integrating long-term adiposity and short-term satiety endocrine signals. The GLP-2R expression has also been reported in extra-hypothalamic areas such as the NTS [58]. The presence of the GLP-2R in other brain areas involved in the regulation of energy balance, including the brainstem (dorsal motor nucleus of vagus nerve [DMV]) and hippocampus (parabrachial neurons), suggests the role of GLP-2 in metabolic regulation [29,57,59,60,61]. Interestingly, GLP-2Rs have been localized on cell bodies of vagal afferents of the nodose ganglion in the rat [62]. GLP-2R signaling in the CNS has been reported to be involved in the regulation of several physiological processes, including feeding behavior and gastrointestinal functions (see below).

**Figure 3:**
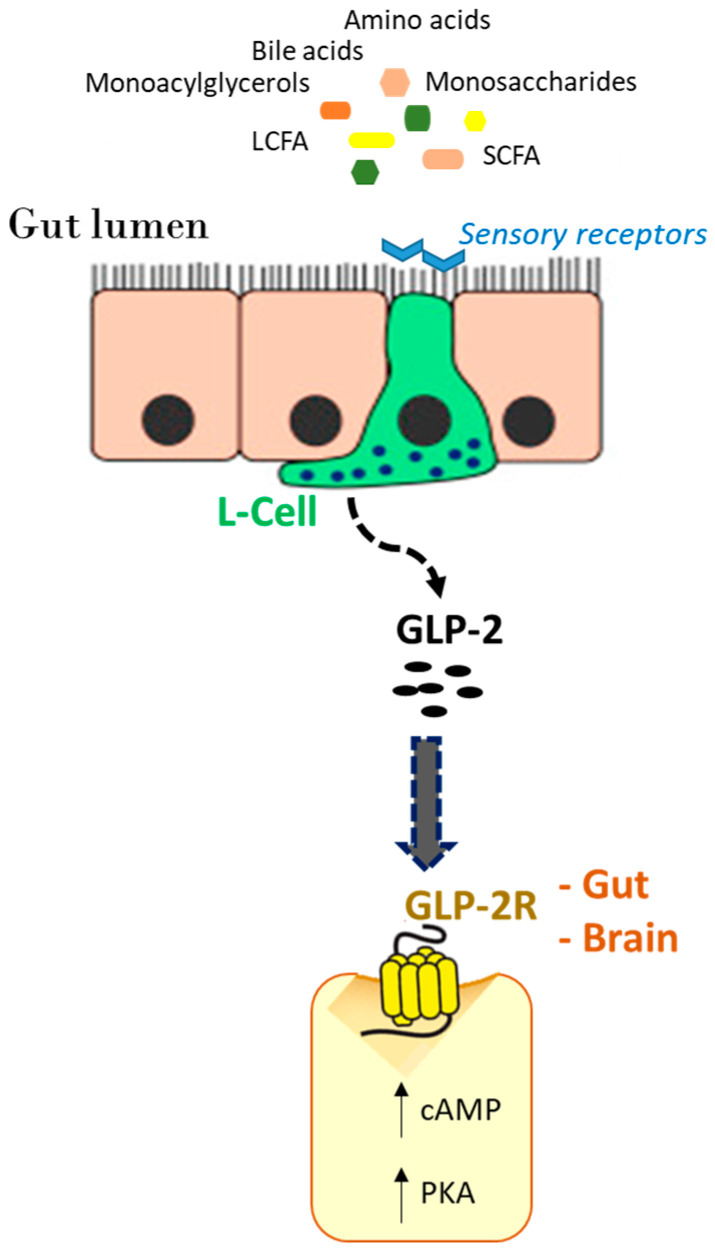
A schematic representation of the activation of the GLP-2R in the gut and brain following the release of GLP-2 from L cells stimulated by nutrients entering the intestinal lumen, which are detected and sensed by receptors on the apical border. SCFA (short-chain fatty acids); LCFA (long-chain fatty acids); cAMP (cyclic adenosine monophosphate); and PKA (protein kinase A).

A schematic representation of the GLP-2R activation following nutrient stimulation of GLP-2 release from L cells is reported in Figure 3.

### Effects of GLP-2 in the Regulation of Food Intake

Among its multiple functions, GLP-2 has also been reported to influence food intake by exerting anorexigenic effects. Mice lacking GLP-2R in POMC neurons showed hyperphagia, supporting a central action of GLP-2 in satiety regulation [60]. Appetite suppression has been observed in mice following activation of the hypothalamic GLP-2R by intracerebroventricular administration of GLP-2 [60] or of a degradation-resistant GLP-2 analog [63]. This hormone effect has been proposed to occur, at least in part, through the activation of the melanocortin receptor-4 (MC4-R) signaling pathway [60]: blockade of MC4R abolished the inhibitory effects on food intake of GLP-2 injection into the NTS in fasted rats has been reported [64]. These observations further support the central inhibitory effects of the hormone on food intake in rodents. Of note, in rodents, the inhibitory effects of GLP-2 on food intake were abolished by the loss of the GLP-1R [65], whereas these effects were increased following the loss of GLP-1R signaling [58]. These results indicate the existence of an interplay between the two hormones in the regulation of food intake. Moreover, inhibition of food intake due to GLP-2 injection in the DMH of fasted rats also occurs through the involvement of a specific GLP-2 signaling pathway: the effects of locally delivered GLP-2 can be blocked by Exendin(9–39), a specific GLP-1 receptor antagonist, but not the MC4-R antagonist SHU9119, revealing that GLP-2 inhibition of food intake in DMH could be blocked functionally by Exendin(9–39) [66]. 

Despite all the reported observations, conflicting results have been found in animals and humans regarding the actual ability of GLP-2 to modulate feeding, and the mechanisms activated by the hormone are still a matter of debate. No influence on appetite or postprandial feeling of satiety has been shown following peripheral administration of GLP-2 in lean, healthy individuals [67,68], whereas inhibition of feeding either by central [56,58] or peripheral [69,70,71] injection of the hormone has been observed in animals. GLP-2 has been also proposed as a neurotransmitter in controlling feeding behavior [69] and may mediate preproglucagonergic neuron-induced synaptic transmission linking the hypothalamus and the brain stem [55].

Although it has been reported that GLP-2 controls energy homeostasis [57], at least in part through the regulation of food intake [72], the involvement of GLP-2 in the hunger-satiety cycle remains subject to debate, and more investigations are certainly required in humans, at variance with the well-established central anorexigenic effects of GLP-1 [73]. Interestingly, the observation that GLP-2 inhibits ghrelin secretion in humans [74] may support the involvement of GLP-2 in the short-term regulation of the hunger-satiety cycle. The effects of GLP-2 on gastrointestinal motility may also indicate its ability to generate peripheral satiety signals from the stomach, which could agree with its central anorexigenic action as detailed in Section 5.

## 4. The GLP-2 Effects in the Gastrointestinal Tract 

### 4.1. GLP-2 and Metabolism

Most of the peripheral regulatory functions of GLP-2 are exerted in the digestive apparatus, where GLP-2R expression has been described [34,61,75,76]. Initially, GLP-2 was identified as an intestinotrophic hormone able to promote the growth and repair of the mouse small intestine [77]. The same effect was later observed in short-bowel jejunostomy patients [78], contributing to the subsequent development of GLP-2 analogs to treat SBS [54]. In the gastrointestinal tract of both humans and animals, GLP-2 signaling modulates the secretion of different enzymes involved in the digestion and uptake of nutrients [46,79,80,81] to control metabolism and promote a positive energy balance [72]. In particular, GLP-2 signaling facilitates the absorption of fatty acids [82], amino acids [83], and glucose [84]. For the latter, peripheral administration of GLP-2 has also been shown to increase the expression of glucose transporters in the mouse small intestine [85]. Moreover, positive effects on glucose metabolism by the hormone have also been observed in obese mice [79]. 

GLP-2 signaling increases dietary lipid absorption, promotes chylomicron release in rodents [82], is implicated in regulating hepatic insulin sensitivity in mice [29], and plays a lipogenic role in the mouse and hamster liver [86]. A particular feature of GLP-2 concerns its ability to regulate lipid handling in the intestine: studies in humans and rodents showed that exogenous GLP-2 not only enhances dietary fat absorption during the postprandial state but also releases intestinally stored lipids during the post-absorptive state through both local and central mechanisms (see [28]). In both these effects of GLP-2, VIP and nitric oxide (NO) have been suggested to be involved [87,88] and, more specifically, an up-regulation of neuronal NOS (nNOS) expression has been observed [89]. Among the multiple GLP-2R signaling pathways [28,35] NO and VIP have been reported to be recruited in many actions of the hormone in the gastrointestinal tract. In this view, GLP-2 has also been reported to increase intestinal blood flow in both healthy humans [90,91] and in patients with SBS [92] by involving NO and VIP. The observation that the hormone-induced enhancement of intestinal blood flow was attenuated in rodents [93] and humans [91] by co-infusion with nitric oxide synthase (NOS) inhibitors proved that this effect was, at least in part, NO-dependent and agrees with the presence of GLP-2R on enteric neurons expressing endothelial NOS [75]. On the other hand, it has been shown that such neurons also express VIP [75] which, as NO, is known for regulating mucosal blood flow. Both these vasoactive neurotransmitters are reported to be important mediators in the increase in blood flow by GLP-2 (see [28]). Furthermore, it has been hypothesized that the increased mesenteric blood flow, following enhanced NO production, might contribute to the increased chylomicron secretion by GLP-2 even if the exact mechanisms through which GLP-2 modulates intestinal lipid handling are still not fully elucidated [28]. 

### 4.2. GLP-2 and Anti-Inflammatory Activity

GLP-2 signaling has also been reported to promote anti-inflammatory functions through the activation of the GLP-2R in human islets [94]. The hormone injection decreased mucosal inflammatory cytokine production in an animal model of enterocolitis [95] while hepatic anti-inflammatory action of GLP-2 was shown in multidrug resistance 2 knockout mice [96] and in obese mice [97]. In the latter, chronic administration reduced inflammation also in the brain [98]. In this view, endogenous GLP-2 has been suggested to exert beneficial effects against some metabolic disorders in both humans and animals [28,72,86,99,100]. The anti-inflammatory effects occurred, at least in part, via VIP release from enteric neurons in a rat animal model of inflammatory bowel disease [101,102,103]. Moreover, GLP-2 was reported to increase the proportion of neurons expressing VIP in cells derived from primary cultures of submucosal enteric neurons [104] similar to what was reported in the colon submucosal plexus of a rat model of colitis [102]. 

In addition to VIP, some of the GLP-2 protective effects have been reported to be mediated by NO. Of note, other than in promoting intestinal growth [105], the NO pathway appears to be involved in the effects of the hormone in preventing cisplatin-induced damage in the gastrointestinal tract of mice: the GLP-2 analog ([Gly(2)]GLP-2) was reported to counteract the morphological and functional damages induced by cisplatin treatment and protect nNOS neurons in mice gastric fundus [106] and distal colon [107]. GLP-2 also attenuates chemotherapy-induced mucositis, reduces epithelial permeability, improves intestinal barrier function, and decreases meal-stimulated gastric acid secretion as well as gastrointestinal motility [108], making the hormone a suitable agent for the treatment not only of SBS but also inflammatory bowel diseases and chemotherapy-induced mucositis [28].

### 4.3. GLP-2 and the Microbiota

Among its different functions, intestinal microbiota has been reported to play a role in the regulation of food intake since some of its metabolites can increase enteroendocrine L cell anorexigenic hormone secretion [27,40]. Alterations in gut microbiota composition have been shown to promote significant changes in satiety signals, acting both locally and via the gut-microbiota–brain axis, likely promoting hyperphagia and thus obesity [40]. Therefore, targeting the gut microbiota might represent a strategy to counteract overweight, in addition to other highlighted beneficial effects, such as improving the activity and efficacy of anticancer drugs [109]. In this view, the protective effects of some bacteria have been reported towards cisplatin in mice experimental models [110]. It has been recently observed that a diet enriched in prebiotics also prevented cisplatin-induced changes in mucus secretion in mice, likely protecting the microbiota [111]. 

Interestingly, gut dysbiosis may also alter gut barrier function, the so-called ‘leaky gut’, which by translocation of pathogens into circulation may represent a contributing cause of obesity-associated systemic inflammation [16,112]. It has recently been reported that chronic enteropathy-related dysbiosis in dogs may contribute to reduced plasma GLP-2 concentrations, suggesting that the association between GLP-2 secretion and microbiome indices may direct future research on the treatment of enteropathies [113]. Thus, the protection of the intestinal barrier integrity exerted by GLP-2 and by substances that counteract dysbiosis could represent an interesting strategy to attenuate inflammation in obesity and its associated comorbidities.

The major functions of GLP-2 on the gastrointestinal tract in both humans and animals are summarized in Table 1, which also includes the effects of the hormone on gastrointestinal motor responses discussed in the following paragraphs.

## 5. Effects of GLP-2 in the Modulation of Gastrointestinal Motility as a Possible Source of Peripheral Satiety Signals Generated by the Stomach through the Gut–Brain Axis 

Several centrally acting hormones can, through efferent innervation, lead to changes in the gastric motor functions known to be involved in the regulation of food intake, such as motility, tone, or emptying [21,122]. Gastric emptying rates can be slowed by the hypothalamus which, through the brainstem nuclei (such as the NTS and the dorsal motor nucleus of the vagus), stimulates vagal efferent fibers that activate intramural gastric nitrergic neurons to decrease gastric motility [18,123]. On the other hand, many hormones that act centrally to influence the hunger-satiety cycle have also been reported to modulate gastric functions and particularly motor responses, even in isolated preparations [19,124,125,126]. These motor responses, in turn, may generate peripheral signals that reach the central structures involved in the regulation of food intake. Therefore, the hunger-satiety cycle may be influenced by a hormone-driven bidirectional gut–brain axis.

Although many studies support both central and peripheral roles for GLP-2 in the regulation of gastric motility, contrasting results have been reported in humans and animals. In pigs and mice, reduced antral motility following intravenous infusion of GLP-2 [115] and suppressed gastric emptying by intracerebroventricular activation of GLP-2R signaling [60] were reported. These hormonal effects were coupled with loss of appetite and occurred, at least in part, through the activation of the MC4-R signaling pathway [60,66]. Interestingly, mice lacking GLP-2R in POMC neurons showed accelerated rates of gastric emptying besides hyperphagia, thus supporting a central role for GLP-2 in either slowing gastric emptying or satiety regulation [60]. Therefore, activation of central GLP-2R appears to play an important role in both the reduction in food intake and gastric emptying rate in rodents. A decreased gastric emptying rate in mice has been reported [69] following peripheral administration of the degradation-resistant analog of GLP-2, [Gly2]GLP2 [35]. The localization of GLP-2Rs on cell bodies of vagal afferents of the nodose ganglion in the rat [62] suggests that GLP-2 from the gut may signal to the hypothalamic nuclei involved in the regulation of food intake, not only directly by crossing the blood–brain barrier, but also through the activation of vagal afferent pathways.

Contrasting results on the ability of GLP-2 to affect gastric emptying have been reported in humans: while peripheral administration of GLP-2 resulted in no effect [67], GLP-2 infusions caused a dose-dependent increase in antral emptying time, although less powerfully than GLP-1 [116]. These discrepant results have been ascribable to the different methodological approaches [33] or to the rapid degradation of peripherally administered native GLP-2 [67,68,69] by the ubiquitous enzyme DPP-IV [127]. 

### GLP-2 and Enteric Nervous System

An important role in the control of gastric motility is played by intrinsic motor neurons, which supply the smooth muscle and whose nervous fibers release a variety of either excitatory or inhibitory neurotransmitters [128,129]. Among them, acetylcholine is known to be one of the major excitatory neurotransmitters released from cholinergic fibers, whereas NO and VIP are considered the main inhibitory neurotransmitters released by non-adrenergic, non-cholinergic (NANC) fibers supplying the smooth muscle and causing gastric relaxation [130,131]. Gastric smooth muscle motor responses are indeed the result of a balance between excitatory and inhibitory nervous activity that may be modulated by hormonal influences. 

The first experimental evidence that GLP-2 can induce gastric relaxation *in vitro* acting on the mouse stomach was provided by Amato and collaborators [117]. Particularly, by recording intraluminal pressure from isolated preparations, they observed that GLP-2 decreased fundus tone, an effect that could actually be regarded as an additional peripheral mechanism contributing to the central anorexigenic actions of the hormone reported in rodents [57]. Notably, inhibition of fundus tone increases murine gastric capacity which may underline the short-term inhibition of food intake by GLP-2 [132]. Furthermore, the decrease in the proximal gastric tone caused by GLP-2 could also delay the flow through the pylorus, thus prolonging the gastric emptying time, as suggested by Amato and collaborators [117]. 

The peripheral effects of GLP-2, through which it modulates rodent gastric fundus motility, are likely due to a neuromodulatory action of GLP-2 on the enteric nervous system. In agreement, GLP-2R protein and mRNA expression was described, other than in enteroendocrine, subepithelial cells and myofibroblasts, in myenteric neurons of humans, rodents, and pigs [75,133,134]. In human preparations, GLP-2R immunoreactivity was detected in the cell bodies of the myenteric neuron but not in the nerve fibers supplying circular and longitudinal muscle layers [134]. In rodents, GLP-2 has been reported to cause gastric fundus relaxation by increasing the release of the two major NANC inhibitory neurotransmitters, VIP and NO. A prejunctional neuronal release of VIP by GLP-2 has been reported to occur in either isolated whole stomachs or gastric circular muscle strips, as proved by the reduced relaxant effects of the hormone following VIP receptor desensitization in mice [117]. Subsequent experiments carried out in the longitudinal strips from mouse gastric fundus showed that the hormone exerted a neuromodulatory action by influencing the component of the neurally induced relaxant responses [119] ascribable to VIP release [131,135]. 

In addition to VIP, GLP-2 has been reported to facilitate smooth muscle relaxation through NO as many other hormones do [118,136,137,138,139]. In fact, the hormone involvement in the circuits that regulate gastric emptying through myenteric inhibitory neurons that release NO has been demonstrated in humans and animals [122,140]. Moreover, in animals, the ability of NO to control the gastric pyloric sphincter, where nitrergic nerves are very numerous [141], has been reported [142], thus supporting its role also in regulating the transit of chyme from the stomach into the duodenum. GLP-2 was found to depress the amplitude of the contractile responses in longitudinal strips from the mouse gastric fundus and to enhance the amplitude of that component of the neurally induced relaxant response [118] ascribable to NO release from NANC inhibitory neurons [143]. Thus, GLP-2 induces proximal stomach relaxation also by modulating the nitrergic neurotransmission, likely through the up-regulation of NO production [143]. Immunohistochemical experiments revealed an increased nNOS immunoreactivity in the nerve structures after GLP-2 exposure of gastric specimens [118]; co-localization of GLP-2Rs with the two constitutive NOS isoforms (eNOS and nNOS) in myenteric and submucosal neurons of the stomach [75]; and colocalization of GLP-2 with NOS or VIP in the myenteric plexus of different mammalian species [144], including human gastric fundus [145].

The above-reported effects of GLP-2 in the stomach may agree with its involvement in the generation of peripheral satiety signals involved in the short-term regulation of food intake. The decrease in the gastric fundal tone by GLP-2, which causes an increased stomach capacity, and the slow gastric emptying rate may be regarded as peripheral mechanisms addressed to suppress food intake. Furthermore, the same decrease in proximal gastric tone caused by GLP-2 would also prolong gastric emptying time.

However, the possible relationship between peripheral effects of GLP-2 on gut motility and the regulation of food intake is not limited to the ability of the hormone to influence motor responses of the stomach since GLP-2 has been proven to exert inhibitory effects on the motility of the small intestine too.

In this view, GLP-2 has been reported to induce changes in intestinal motor responses acting either centrally or peripherally, through a dual mechanism involving the inhibition of the excitatory component and/or an enhancement of the inhibitory one. GLP-2 administration *in vivo* has been shown to inhibit intestinal transit in mice [146], likely by exerting a neuromodulatory role to increase inhibitory inputs on excitatory enteric neurons. This mechanism fits well with the detection of GLP-2R expression on either excitatory or inhibitory myenteric and submucosal neurons of the mouse duodenum, in which the hormone depressed contractile responses by decreasing the cholinergic neurotransmission and by increasing NO production in isolated preparations [76]. 

More recently, it has been demonstrated that GLP-2 also depresses ileal contractility in isolated preparations from mice [120], thereby filling a gap in the literature on the effects of the hormone in this isolated intestinal segment where its production occurs and in which GLP-2Rs are also highly expressed [147]. The depressant effects of GLP-2 on ileal contractility likely occur through a dual opposite modulatory effect on inhibitory nitrergic and excitatory cholinergic neurotransmission, as supported by immunohistochemical results showing a significant increase in nNOS-positive fibers in the ileal muscle wall and a significant decrease in ChAT-positive myenteric neurons in GLP-2-exposed preparations [120]. 

**Figure 4:**
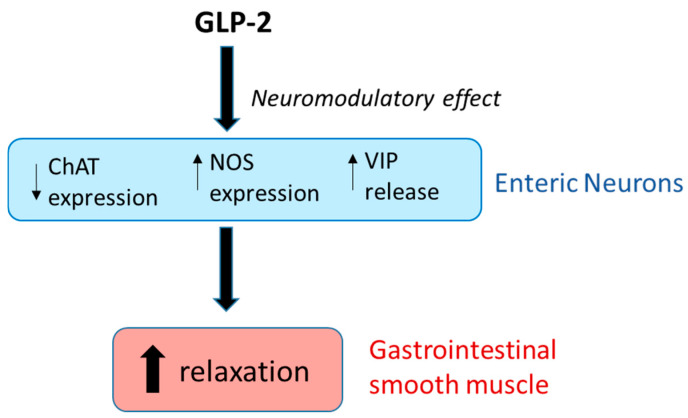
Schematic illustration summarizing the main modulatory actions of GLP-2 on the enteric neurotransmission. GLP-2 decreases the number of choline acetyl transferase (ChAT)-positive myenteric neurons, reducing the excitatory cholinergic input to the smooth muscle. The indirect pro-relaxant effects of GLP-2 are also exerted through a modulatory action on NANC inhibitory neurotransmission. GLP-2 modulates the nitrergic neurotransmission by up-regulating nitric oxide synthase (NOS) expression, thereby increasing NO production/release. GLP-2 also enhances VIP release from myenteric neurons.

A simplified scheme summarizing the neuromodulatory action of GLP-2 on enteric neurons is reported in Figure 4.

The general physiological significance of the depressant effects of the hormone on intestinal motility could be directed at prolonging the transit time and thus promoting nutrient absorption processes. The same is true for the depressant action of GLP-2 on ileal contractility that extends the permanence of the contents in the more proximal intestinal portions. This function agrees with the reported role of the hormone in the small intestine [34] and the successful introduction of a GLP-2 analog in the treatment of patients affected by SBS [54]. However, GLP-2 favoring nutrient absorption would even appear in contrast with its anorexigenic effects, which should have an impact on body weight loss. On the other hand, the inhibitory effects of GLP-2 on intestinal contractility by increasing the contact time of nutrients with enteroendocrine cells may increase anorexigenic hormone release, as occurs in the ileal brake reflex [148,149], also generating a positive feedback loop on its own release. Moreover, as reported in the introduction section, the activation of the ileal brake reflex leads to delayed gastric emptying. Therefore, even if many aspects still need to be clarified, the inhibitory effects exerted by GLP-2 on small intestine contractility could indeed represent an additional peripheral satiety signal in rodents. The effects of GLP-2 on gastrointestinal motor responses, some of which may agree with its role in the generation of peripheral satiety signals, are reported in Table 1. 

## 6. Concluding Remarks 

The increasing incidence of obesity has led to a growing interest in the gastrointestinal tract as a potential target for both pharmacological and nutritional approaches to weight management [150]. In this context, the role of gut-derived hormones in the gut–brain axis is a topical issue in the regulation of food intake. 

While the role of some gut-derived hormones to inhibit food intake not only centrally but also peripherally by inducing changes in the gastrointestinal motor responses has been recognized in humans, this relation for GLP-2 has not been fully elucidated yet. Nevertheless, among its several effects, GLP-2 has been reported to exert central anorexigenic actions in animal preclinical studies and to affect those gastrointestinal motor responses whose changes are strictly related to the generation of peripheral satiety signals through the gut–brain axis. These peripheral effects may represent an additional mechanism engaged by the hormone, contributing to its central actions, in the short-term regulation of food intake in rodents. Therefore, a better understanding of this mechanism could point towards a possible use of GLP-2 analogs as an additional strategy for the management of body weight gain. In this view, GLP-2R has been recently proposed as a target for the treatment of obesity [16]. Moreover, it has been hypothesized that an altered intestinal microbiota, which is primarily affected by diet composition, may contribute to the onset of obesity through the gut-microbiota–brain axis [151]. Intestinal microbiota metabolites have been reported to have a role in inducing the release of GLP-2 as well as other anorexigenic hormones by intestinal enteroendocrine cells [27,40]. Although microbiome manipulation for the treatment of obesity needs to be further explored in humans, diet, prebiotics, probiotics, and symbiotics may have a beneficial impact on several metabolic pathway disorders involved in the onset of obesity [152].

Among the new proposed therapeutic approaches, stimulation of the endogenous secretion of glucagon-like peptides from enteroendocrine L-cells has been suggested [27] given the successful introduction of GLP-1-based drugs in the treatment of obesity. From this perspective, a possible future scenario in the approach to the treatment of obesity could be to exploit the different systems present in enteroendocrine cells that are affected by substances that modify the release of hormones. A better understanding of the mechanisms by which GLP-2 may influence food intake may therefore be important for its therapeutic implications beyond its clinical use for the treatment of SBS. However, limitations in its therapeutic applications should be considered, mainly due to its short half-life and possible side effects also related to the pharmacological activation of the GLP-2R [16].

## Figures and Tables

**Table 1:** Effects of GLP-2 signaling on the gastrointestinal tract.

Effects of GLP-2 Signaling	Route of Administration	Species	References
Increased small intestinal weight and jejunal crypt-villus height	Subcutaneous	Mouse	[77]
Increased villus height and crypt depth	Subcutaneous	Human	[78]
Increased jejunal amino acid absorption	Isolated preparations	Mouse	[83]
Increased glucose uptake	Intravenous	Piglet	[84]
Increased expression of glucose transporters	Peripheral administration	Mouse	[85]
Increased fatty acids absorption	Intraperitoneal	Mouse and hamster	[82]
Increased plasma levels of free fatty acids and triglyceride	Intravenous	Human	[80]
Increased plasma levels of chylomicron and triglyceride	Subcutaneous	Human	[91]
Mobilization of intestinally stored lipids	Intraduodenal	Mouse and hamster	[87]
Mobilization of intestinally stored lipids	Intraperitoneal	Rat	[114]
Decreased mucosal inflammatory cytokine production	Subcutaneous	Rat	[95]
Reduction in pro-inflammatory cytokines and crypt cell apoptosis	Subcutaneous	Rat	[101]
Increased intestinal blood flow	Intravenous	Human	[90]
Increased intestinal blood flow	Subcutaneous	Human	[91]
Increased intestinal blood flow	Subcutaneous	Human	[92]
Increased intestinal blood flow	Intravenous	Piglet	[84]
Increased intestinal blood flow	Jugular vein	Rat	[93]
Prevention of cisplatin-induced morphological changes in the gastric fundal strips	Intraperitoneal	Mouse	[106]
Prevention of cisplatin-induced morphological changes in isolated distal colon	Intraperitoneal	Mice	[107]
Reduced antral motility	Intravenous	Pig	[115]
Gastric emptying inhibition	Intracerebroventricular	Mouse	[60]
Gastric emptying inhibition	Central	Human	[116]
Decreased gastric emptying rate	Peripheral	Mouse	[69]
Gastric smooth muscle relaxation	Isolated whole organ	Mouse	[117]
Gastric smooth muscle relaxation of fundal strips	*In vitro*	Mouse	[117]
Increased amplitude of the neurally induced relaxation of gastric fundal strips	*In vitro*	Mouse	[118,119]
Counteracted cisplatin-induced increase in the amplitude of contractions in the gastric fundal strips	Intraperitoneal	Mouse	[106]
Inhibition of duodenal contractions	Isolated whole preparation or segment	Mouse	[76]
Depression contractility of ileal segments	*In vitro*	Mouse	[120]
Inhibition of contractility in isolated colonic segments	*In vitro*	Mouse	[121]

## Abbreviations

AgRP	Agouti-related protein
CART	cocaine- and amphetamine-regulated transcript
CCK	cholecystokinin
ChAT	choline acetyl transferase
CNS	central nervous system
GLP-1	glucagon-like peptide-1
GLP-2	glucagon-like peptide-2
GLP-2R	glucagon-like peptide-2 receptor
NANC	non-adrenergic, non-cholinergic
NO	nitric oxide
nNOS	neuronal nitric oxide synthase
NPY	neuropeptide Y
NTS	nucleus tractus solitarius
POMC	pro-opiomelanocortin
PYY	peptide tyrosine tyrosine
SBS	short bowel syndrome
VIP	vasoactive intestinal peptide
